# Supplementary material for: Single-Cell Transcriptomics Reveals the Complexity of the Tumor Microenvironment of Treatment-Naive Osteosarcoma
Source: Front Oncol. 2021 Jul 21;11:709210. doi: 10.3389/fonc.2021.709210 (PMC8335545; doi:10.3389/fonc.2021.709210)
Supplement: Supplementary Document 1 — Kaplan-Meier curve of the 518 genes associated with survival. [file DataSheet_1.zip › Supplementary Document 1/sur.PKHD1L1.pdf]

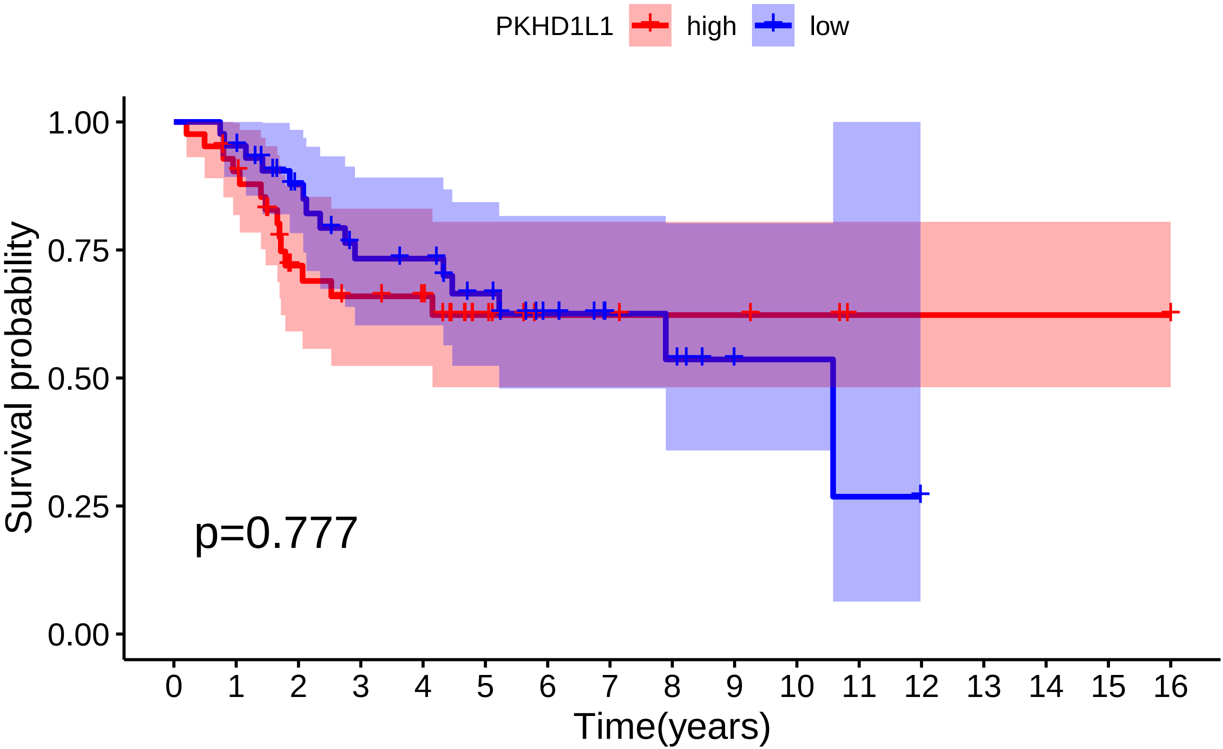

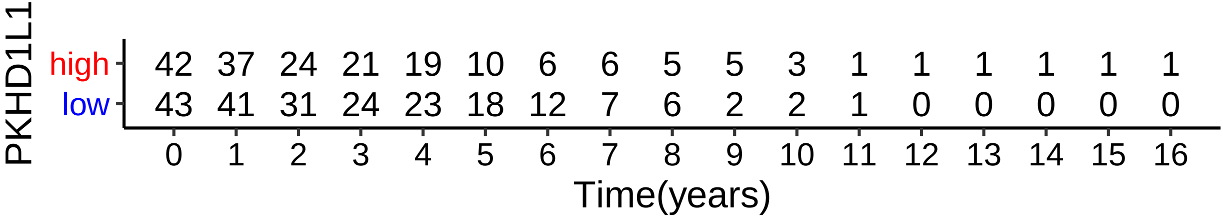

PKHD1L1	0	1	2	3	4	5	6	7	8	9	10	11	12	13	14	15	16
high	42	37	24	21	19	10	6	6	5	5	3	1	1	1	1	1	1
low	43	41	31	24	23	18	12	7	6	2	2	1	0	0	0	0	0
